# Evolutionary and methodological considerations when interpreting gene presence–absence variation in pangenomes

**DOI:** 10.1093/nargab/lqag011

**Published:** 2026-01-30

**Authors:** Tomáš Brůna, Avinash Sreedasyam, Avril M Harder, John T Lovell

**Affiliations:** DOE Joint Genome Institute, Lawrence Berkeley National Laboratory, 1 Cyclotron Road, Berkeley, CA 94720, United States; DOE Joint Genome Institute, Lawrence Berkeley National Laboratory, 1 Cyclotron Road, Berkeley, CA 94720, United States; Genome Sequencing Center, HudsonAlpha Institute for Biotechnology, Huntsville, AL 35806, United States; Genome Sequencing Center, HudsonAlpha Institute for Biotechnology, Huntsville, AL 35806, United States; DOE Joint Genome Institute, Lawrence Berkeley National Laboratory, 1 Cyclotron Road, Berkeley, CA 94720, United States; Genome Sequencing Center, HudsonAlpha Institute for Biotechnology, Huntsville, AL 35806, United States

## Abstract

While graph-based pangenomes have become a standard and interoperable foundation for comparisons across multiple reference genomes, integrating protein-coding gene annotations across pangenomes in a single ‘pangene set’ remains challenging, both because of methodological inconsistency and biological presence–absence variation (PAV). Here, we review and experimentally evaluate the root of genome annotation and pangene set inconsistency using two polyploid plant pangenomes: cotton and soybean, which were chosen because of their existing diverse high-quality genomic resources and the known importance of gene PAV in their respective breeding programs. We first demonstrate that building pangene sets across different genome resources is highly error prone: PAV calculated directly from the genome annotations hosted on public repositories recapitulates structure in annotation methods and not biological sequence differences. Re-annotation of all genomes with a single identical pipeline largely resolves the broadest stroke issues; however, substantial challenges remain, including a surprisingly common case where exactly identical sequences have different gene model structural annotations. Combined, these results clearly show that pangenome gene model annotations must be carefully integrated before any biological inference can be made regarding sequence evolution, gene copy-number, or PAV.

## Introduction

Pan-genome references, or the representation of all available DNA sequences across groups of organisms in a single integrated resource (hereon ‘pangenomes’), are rapidly becoming the standard foundation for genetics research in medicine, agriculture, biotechnology, ecology, and evolution [1]. This paradigm shift has been made possible by advances in long-read sequencing technology and assembly algorithms. Multiple functionally complete genomes are now available for many species.

The tools to construct, compare and visualize pangenomes have also rapidly advanced in a handful of heavily invested model systems. In these cases ‘graph’ representations have been shown to improve variant detection [2–4], providing clues to evolutionary processes and sequence diversity in previously inaccessible regions of the genome. In contrast to the technological advances that aid in the construction and integration of multiple nearly complete and consistent genome assemblies, *de novo* annotation of putatively functional protein coding gene models remains challenging and expensive [5, 6].

Typically, protein-coding gene annotations are guided by one of three main data types: (i) ‘homology’ similarity to well-supported but phylogenetically distant protein sequences, (ii) ‘*ab initio*’ gene structure evidence, or (iii) short-read and full-length RNA sequencing-based ‘transcriptomes’ [7]. However, each of these evidence structures can produce high error rates: (i) homology-driven methods are unlikely to discover true new genes or gain-of-function variants (high false negative), (ii) *ab initio*-driven methods tend to have high rates of false-positive ‘artifact’ genes, and (iii) transcriptome-driven methods often produce variable results depending on the amount, type, and source (but see below) of sequencing support.

Recently, Gabriel *et al.* [8] demonstrated that ‘integrative’ annotation methods, which expend the substantial compute and sequencing costs to combine the three main evidence supports (homology, *ab initio*, and transcriptome), are likely to best discover true gene presences and minimize false positives. However, given the costs of integrative pipelines, many annotations of pangenomes now heavily rely on *ab initio* and/or homology support (e.g. Helixer [9] or Galba [10]). In the extreme case, some pangene sets are generated from gene model propagation ‘lift-over’ from a single reference to all members of the pangenome (e.g. Liftoff [11]). However, annotation methods that rely solely on lifting reference gene models are incapable of new gene discovery and are therefore not appropriate for any but the most homogenous pangenomes, which are probably best represented by a single-haplotype linear reference genome.

Individual gene annotation quality is typically assessed through complementary metrics: completeness measures such as BUSCO [12], which quantify the presence of expected conserved orthologs as true-positives, and precision measures such as PSAURON [13], which estimate false-discovery rates by identifying spurious predictions. Because genomic inquiries in many systems are beginning to rely on multiple references, consistency of gene annotations for orthologous sequences across references is a third and particularly important quality metric. Such consistency is critical for evolutionary inference and practical applications of pangenomes: often the most important product from a pangenome is functional sequence variation across a set of genes (i.e. the ‘pangene set’) that share an evolutionary common ancestor (i.e. pairwise ‘orthologs’ or sets of genes in ‘orthogroups’).

When approaching pangenome annotations, these three aspects—completeness, precision, and consistency—must be considered and balanced. The most consistent annotations (e.g. lift-overs) will by definition be unable to find new genes and thus suffer from false-negatives in gene lists. Conversely, highly sensitive approaches that identify all possible CDS typically generate high false discovery rates in individual genomes and correspondingly low consistency. However, the extent to which pangene sets are consistent, both across annotation methods and within pangenomes annotated by identical approaches, is not well understood.

In this study, we investigate the extent to which gene presence–absence variation (PAV) in pangenomes is driven by biological versus methodological factors, with particular attention to the challenges of *post hoc* integration of gene annotations generated with diverse computational methods and input resources. We first examine PAV patterns in plants and animals across both recently to anciently divergent phylogenetic contrasts, which reveals that PAV is strongly related to divergence time in both taxonomic groups, but much more prevalent in plants than animals. We then analyze publicly available cotton and soybean pangenomes to quantify how annotation methodology impacts inferences of relatedness and gene content variation. Our results demonstrate that while biological factors certainly contribute to gene PAV, methodological inconsistencies can dramatically inflate estimates of pangenome “openness” and potentially mislead evolutionary and functional interpretations. These findings have important implications for pangenome construction, annotation strategies, and downstream applications in breeding, comparative genomics, and evolutionary biology.

## Materials and methods

### Comparison of divergence time and orthology

RefSeq genome annotations were downloaded from NCBI at URLs reported in Supplementary Table S1 (amniote animals) and Supplementary Table S2 (vascular plants). OrthoFinder was run on each set independently, and pairwise orthologs were extracted from the resulting output. For each pair of genomes, all genes were categorized as: (i) one-to-one (1:1) orthologs where only a single gene was present in the orthology table for each genome, (ii) PAV as genes without an ortholog, or (iii) CNV as orthologs with different numbers of genes in each genome. If none of these were satisfied, the genes were multicopy orthologs. These orthology categories were merged with pairwise estimates of divergence time, downloaded from timetree.org (Supplementary Data 1).

### Genome resources

We analyzed publicly available pangenome resources for two polyploid crop species: soybean (*Glycine max; n* = 37 genomes) and upland cotton (*Gossypium hirsutum; n* = 7 genomes). These genomes were produced by multiple independent annotation consortia, and the soybean dataset included three independently assembled versions of cultivar ‘Wm82’ and two of ‘Zh13’. All original genome assemblies and annotations were downloaded from SoyBase [14] and CottonGen [15], respectively. Full details of each genome assembly and annotation source are provided in Supplementary Tables S3 and S4.

### Assembly-based assessments of pangenome openness and similarity

To assess assembly-based pangenome openness, we used Pangrowth (v1.0.0) [16] which calculates an exact growth curve based on a PAV matrix for all 31-mers in the input assemblies. To estimate similarity among assemblies in both sets, we used PanKmer (v0.20.4) [17] to calculate an adjacency matrix based on the number of shared 31-mers between all pairs of genomes. We converted these adjacency values to Jaccard similarity values for assembly clustering and heatmap generation.

### Annotation-based assessments of PAV, similarity, and openness

Original (a.k.a., ‘raw’) annotations were downloaded from public repositories including Soybase and CottonGen, and NCBI’s genome portal. The US Department of Energy Joint Genome Institute’s (JGI) Integrated Gene Calling pipeline (IGC; see below for details) was applied to all genomes. For both the ‘raw’ and IGC annotations, a single ‘primary’ transcript was selected for each gene model, defined as the transcript with the longest CDS. Gene presence–absence and copy-number variation was determined by membership in OrthoFinder [18] defined phylogenetically hierarchical orthogroups (a.k.a. ‘HOGs’), implemented through GENESPACE [19].

### Consistent re-annotation with JGI’s IGC pipeline and Helixer

Primary analyses in this study used annotations generated by the IGC pipeline, the JGI’s internal annotation method. IGC has previously been used to annotate much of JGI’s large-scale plant genome work, including the reference soybean genome and most genomes hosted on Phytozome. Because it is tightly integrated with JGI’s high-performance computing infrastructure and licensed resources, IGC is not currently available for public release, though efforts toward a portable version are underway.

Seven cotton genomes were annotated with IGC following the same protocol described in [20]. For each cotton genome, we used the same RNA–Seq dataset that was employed in its original annotation (Supplementary Table S4). All soybean genomes were annotated using the same protocol, but each employed a common transcriptome dataset generated for the reference Wm82.a6.v1 genome [21]. Gene annotations were also generated using Helixer [9, 22] with land_plant_v0.3_a_0080.h5 parameters.

### Assessing annotation consistency

To evaluate annotation consistency between any two genomes (Query and Target), we aligned the predicted coding sequences (CDS) from the Query genome to the Target genome using Liftoff [11]. A CDS was considered *perfectly mapped* if it aligned with 100% sequence identity over its full length, with no gaps or mismatches. The resulting mapped gene structures were compared to the Target genome annotation using gffcompare [23] in the strict mode. We calculated two metrics: *consistency*, defined as the proportion of *perfectly mapped* CDS whose genomic coordinates either exactly matched or were fully contained within those of a single predicted gene in the native annotation of the Target genome on the same strand; and *overlap*, defined as the proportion of perfectly mapped CDS that overlapped by at least one base pair with any predicted gene in the native annotation of the Target genome on the same strand, regardless of coordinate match or containment.

## Results and discussion

### What is the scale of gene PAV across annotations?

The relative degree to which two genome annotations are ‘consistent’ depends largely on two factors: (i) the *biological* predisposition of genomes to lose or gain true protein-coding genes, and (ii) the *technical* ability for annotation method to identically call similarly structured genes models from orthologous DNA sequences. Biological causes of gene PAV abound in the literature (e.g. [24, 25]), and it has been hypothesized that lineages that have undergone whole-genome duplications, like most plants, fungi [26], insects [27], but not most vertebrates (see [28]), will harbor biologically relevant gene copy number variation (CNV) and PAV that may drive speciation and complex trait evolution [29].

**Figure 1.**
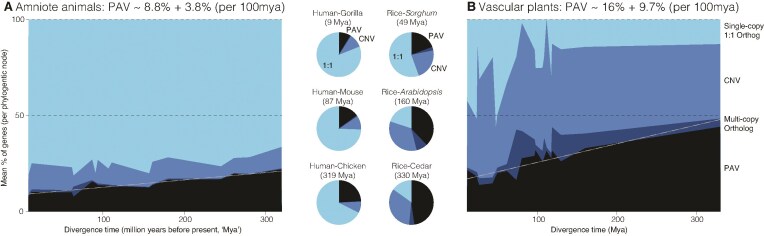
CNV and PAV between pairs of vascular plant and amniote genomes. Pairwise orthologs (defined by OrthoFinder) were calculated separately for NCBI RefSeq protein-coding gene annotations across the diversity of amniotes (**A**, *n*_mammals_ = 7, *n*_birds_ = 4, *n*_other reptiles_ = 9) and vascular plants (**B**, *n*_eudicots_ = 15, *n*_monocots_ = 7, *n*_gymnosperms_ = 1) and grouped into four orthology classifications: one-to-one (‘1:1′) orthologs, presence–absence variants (‘PAV’, ortholog missing in one genome), copy-number variants (‘CNV’, different CNV between genomes), or multicopy orthologs. The area charts show the mean proportion of these four categories for each unique internal node in a time-calibrated phylogenetic tree produced by timetree.org. The linear model of mean PAV% as a function of divergence time is plotted as a white dashed line and the model coefficients are reported in the panel headers. The pie charts present these values for three contrasts of important genomes representing the closest and most diverged plant and animal genomes analyzed here.

We tested the extent that gene PAV and CNV vary across two taxonomic groups (vascular plants, amniotic animals) with divergence times ranging from ∼10M to > 300M years (Fig. 1 and Supplementary Data 1). Unsurprisingly, within both vascular plants and amniotes, gene PAV becomes increasingly common with increasing divergence time—more diverged genomes share fewer orthologs, likely due to sequence deletions, pseudogenization, and other forms of large-scale mutation followed by selection or drift. However, the absolute amount of gene PAV and the degree that PAV is affected by divergence time was quite different between plants and animals. For example, the most closely related plant contrast that does not span a whole genome duplication (∼49 Mya *Setaria*-*Sorghum* split) exhibited similar PAV (4758 genes, 16.9% PAV) as the 319Mya diverged human and chicken genomes (3412 genes, 18.9%). Overall, vascular plant genomes exhibit nearly twice the base level of gene PAV, and the degree of PAV increases at more than double the rate with corresponding increased divergence time, compared to amniotes.

Single copy orthologs were also far more common in amniotic vertebrates than vascular plants. For example, the two most common flowering plant model systems (*Arabidopsis thaliana* and rice, ∼160Mya divergence) have only 20.6% of their genes in 1:1 orthologs, while human and chicken genomes share 72.1% of their genes as single copy orthologs. This is consistent with previous observations [30], which attributed the paucity of 1:1 orthologs to pervasive nested whole genome duplications across the two major flowering plant lineages.

In these analyses, we strove to reduce technical artifacts by ensuring that all annotations were generated by the NCBI RefSeq pipeline. Therefore, most variation in ortholog classifications compared in Fig. 1 should be related to biology and not differences among annotation methods. However, it is fairly common to calculate and assign biological significance to PAV defined by different annotations. Comparisons among completely different annotation methods have been shown to introduce an abundance of ‘orphan’ genes in *post-hoc* integrated pangene sets [31]. However, it is not clear whether annotation methods that are nominally similar (e.g. all being “integrative” approaches that combine multiple evidence types) will produce similar false positive results, particularly within species where genomic sequences are of high quality and per base sequence diversity is low. Understanding these effects is critical to accurately interpret pangenome statistics, as methodological inconsistencies could easily be misinterpreted as biologically meaningful variation among closely related genomes.

### Cotton and soybean as model systems for studying annotation-derived PAV

Combined, the analyses across vascular plants and amniotes clearly demonstrate the biological importance of gene PAV, even among closely related plant genomes. Given the clear predisposition of plant genomes to evolve PAV, we opted to focus on angiosperm pangenomes as a case study for the relative impacts of methodology on inference of gene PAV.

To test the relative impacts of methods and sequence evolution, we compared gene PAV in two aggregated sets of integrative annotations: soybean (*n* genomes = 37 , Supplementary Table S3) and cotton (*n* genomes = 7, Supplementary Table S4). Like sets of reference genomes for most species, these resources were not built in one coordinated effort. Instead, these cotton and soybean genomes were respectively built and published by several groups of scientists, each using their own methodologies (hereon ‘consortia’). This provides an excellent opportunity to examine how different methodologies affect the inference of pangenome variability. Moreover, these crops are ideal for our analysis because their limited genetic diversity (due to domestication bottlenecks) provides a controlled background against which methodological effects can be more clearly distinguished from biological variation.

Despite being constructed using different sequencing approaches and computational methods, the genome assemblies in these datasets are highly contiguous (Supplementary Tables S3 and S4). Each consortium also generated integrated annotations by incorporating the three major lines of evidence (homology, *ab initio* prediction, and transcriptome data), with all cotton and two soybean genomes having native RNA-seq from the identical genotype used for assembly. Importantly, each individual annotation is of high quality based on standard gene prediction evaluation metrics measuring completeness (BUSCO cotton/soybean: mean = 98.0/96.7%, standard deviation (SD) = 1.15/1.7%) and precision (PSAURON cotton/soybean: mean = 94.6/93.4%, SD = 1.8/0.74, Supplementary Table S3 and S4) with the exception of Zh13 [32], which is an older annotation with conspicuously low BUSCO scores. This combination of high-quality assemblies with independently-generated annotations makes these datasets ideal for distinguishing methodological from biological sources of gene PAV.

### Annotation artifacts dominate biological phylogenetic signals in pangenome gene analyses

**Figure 2.**
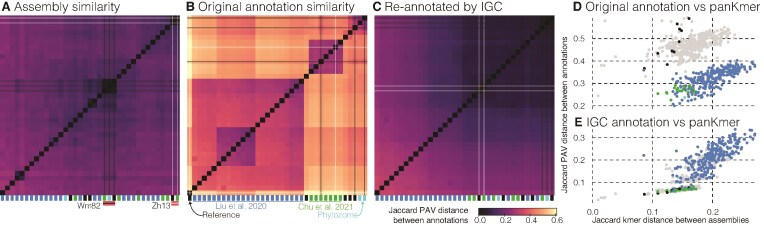
Clustering by annotation and assembly similarity reveals that original gene PAV is driven almost exclusively by annotation method in soybean. Distance matrices between soybean varieties were calculated from PanKmer (**A**) and OrthoFinder gene PAV of the originally published (**B**) and IGC (**C**) protein-coding gene annotations. In each panel, distances are hierarchically clustered to group the most similar genomes together. Lower values (darker colors) indicate greater similarity between genomes. The annotation consortium is flagged below each column in the matrices using colored bars: Liu *et al.* [33] (dark blue), Chu *et al.* [34] (green), Reference genomes annotated independently (black), and Phytozome (light blue). As a proof of concept, we analyzed redundancy among the pangenome members, which contain three versions of ‘Wm82’ (black lines) and two of Zh13 (white lines). Clear clustering of these nearly identical sequences in k-mer space, but not in annotation PAV similarity confirms the major role that gene annotation method plays when inferring biological roles of gene PAV. Correlations between the PanKmer Jaccard distance and the original raw (**D**) and single-method IGC (**E**) PAV distances are presented where points are colored following panel B for comparisons within consortia, with gray indicating comparisons between different consortia.

Genome-wide gene PAV should largely mirror phylogenetic relationships inferred from single copy DNA or protein sequence alignments, despite very different mutational dynamics. For example, soybean germplasm exhibits strong population structure, and Liu *et al.* [33] specifically sampled a wide range of diverse ‘landrace’ locally-adapted cultivars. As expected, sequence-based k-mer distances reveal the expected evolutionary relationships among soybean genomes, with separation of cultivars based on their known genetic relationships. Furthermore, this method clearly groups the replicated nearly identical ‘Wm82’ (*n* = 3, black horizontal/vertical lines) and ‘Zh13’ (*n* = 2, white lines) assemblies (Fig. 2A); genomes of these fully inbred cultivars should have nearly identical sequences due to the seed increase via selfing.

However, a fundamentally different pattern emerged when we clustered genomes based on gene PAV using existing annotations hosted on SoyBase [14]. Rather than clustering by evolutionary relatedness, genomes clustered into four groups almost exclusively defined by which consortium produced the annotation (Fig. 2B). This methodological signal was so strong that it entirely obscured the true biological relationships among the soybean genomes. Furthermore, the Wm82 and Zh13 assemblies annotated by different groups were more distant from each other than those from different genotypes annotated by the same consortium. We observed similar patterns in cotton genomes downloaded from CottonGen [15], though the effect was less pronounced because the particular cotton cultivars included in our study show less clear separation based on sequence and we do not have replication of assemblies with different annotation methods (Supplementary Fig. S1).

Given that annotation methods strongly influenced PAV-based clusters, it is likely that differences between the methods themselves are the driving factors. To test this hypothesis, we re-annotated all genomes using the JGI IGC pipeline. The resulting PAV-based trees successfully recovered the known evolutionary relationships, now closely mirroring the sequence-based trees (Fig. 2C). Critically, while assembly-based k-mer distance is completely unpredictive of PAV distance from naïve integration of the original annotations (Fig. 2D; one-sided Mantel *r* = −0.12, *P* = 0.926), sequence divergence was a strong predictor of PAV from re-annotation by a consistent method (Fig. 2E; one-sided Mantel *r* = 0.861, *P* <.001). This result demonstrates that consistent annotation methodology is essential to accurately capture biological signals in pangenome analyses.

### Variation in consistency among annotation methods is a major driver of estimates of pangenome ‘openness’

Biological pangenomes (i.e. all of the unique sequence present in a species) are typically classified as either ‘open’ or ‘closed’ based on how gene or sequence content changes as additional genomes are analyzed [35]. In a closed pangenome, the number of new genes discovered approaches zero as more genomes are added, suggesting a finite gene repertoire. In contrast, an open pangenome continues to yield new genes with each additional genome, suggesting an unbounded gene repertoire; however, the degree of openness is relative and there is no clear method to quantify the absolute degree of openness across species. This classification has implications for understanding species’ evolutionary potential, genomic flexibility, and breeding strategies, as it influences estimates of untapped genetic diversity and the likelihood of discovering novel beneficial alleles.

**Figure 3.**
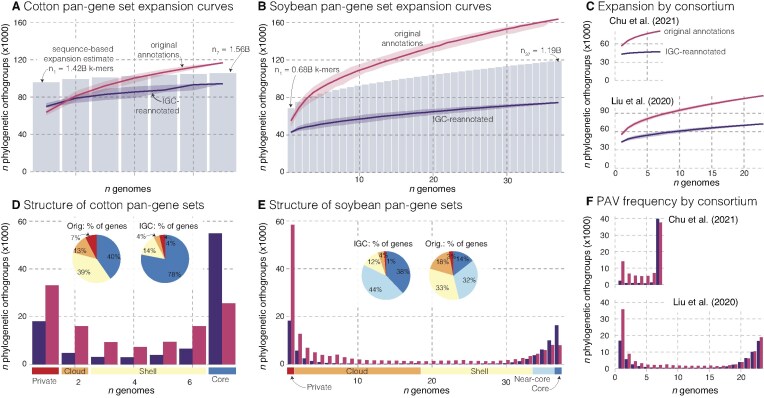
Pangenome expansion and structure in 11 cotton and 37 soybean genomes. (**A**) Cotton pangene set expansion curves, which depict the number of orthogroups for a given number of genomes, are presented for the original (red) and IGC-re-annotated (blue) gene sets. Solid lines and transparent polygons show mean and 95% confidence intervals of 500 random genome-ordering simulations. The gray bar chart gives a baseline for pangenome expansion based on patterns of shared k-mers among assemblies and is standardized so that the total number of k-mers observed is the midpoint between original and IGC curve maxima (see labels for the amount of sequence represented by the smallest and largest bars). (**B, C**) Follow panel (A) but for the 37 soybean genomes and annotations, and for the seven and 23 annotations by Chu *et al.* [34] and Liu *et al.* [33], respectively. (**D**) Cotton pangenome composition as a function of orthogroup PAV, categorized as private (found in a single genome), cloud (<50% of genomes), shell (<90%), near-core (≥90%), and core (found in all genomes) categories for both original (red) and IGC-re-annotated (blue) gene sets. Inset pie charts show the proportion of genes in each category by annotation type. (**E**) Follows panel (D) but for the 37 soybean genomes and annotations. (**F**) Follows panel (E) but for the seven genomes published in Chu *et al.* and the 23 in Liu *et al.*

Building on our finding that annotation methodology overwhelms biological signals in phylogenetic analyses, we investigated how annotation inconsistencies affect gene PAV estimates among genomes. The genetic bottleneck followed by rare introgressions from related species in both cotton and soybean [21, 36] provides an ideal setting to analyze the openness of a pangenome: while most sequence should be highly similar, low-frequency highly diverged sequences associated with adaptive introgressions are crucial targets for breeders. Furthermore, the genetic diversity present in Liu *et al.*’s [33] wild and landrace genotypes should provide more opportunity to explore low-frequency gene PAV in soybean. Estimates of pangenome sequence expansion from k-mers were consistent with this situation: initially the pangenomes expanded by 48/66M k-mers (3.4/9.6%) per cotton/soybean genome, whereas, after 10 genomes were added, expansion was limited to 8.3/12.7M k-mers (0.5/1.4%, Fig. 3A) per genome on average. Combined, these pangenomes are not completely closed, but each genome after the first 10 appear to add proportionally very few new alleles.

In contrast to a closed sequence-based pangenome estimate, when examining pangenome growth using existing annotations, both cotton and soybean pangenomes appeared completely open, with seemingly unlimited expansion potential. For example, when using OrthoFinder’s [18] HOGs, each cotton and soybean genome on average contributed 8851 (11.5% of the average number of genes in an annotation) and 3006 (5.3%) new HOGs, respectively (Fig. 3B).

However, consistent re-annotation with a single approach dramatically transformed this picture: after annotation with the JGI IGC pipeline, each additional cotton genome contributed only 4008 unique HOGs (5.5% of average), while each soybean genome added just 887 HOGs (1.9% of average)—reductions of ∼55% and ∼70% respectively in the rate of pangenome expansion relative to the original annotations. This IGC-reannotated pattern of pangenome expansion much more closely mirrors that of sequence-based estimates (gray bar charts in the background, Fig. 3A and B). Combined, it is clear that expansion statistics, including the ‘openness’ of a species pangenome, follow a similar pattern as genetic distance estimates: naïve integration across gene families calculated from original gene annotations produces pangenome statistics that are wildly different from sequence based estimates. Such artifacts are mostly resolved by using a consistent annotation method.

Given the massive differences between single-method and multimethod annotation integration results (Fig. 2B and C), we assumed that the bulk of artifactual pangenome expansion would be due to differences among annotation methods. Indeed, each annotation method did produce many HOGs that are only found in that one consortium. For example, among the 81 104 HOGs in the Chu *et al.* [34] annotations, 15 727 (19.4%) are only ever found in those seven genomes and never in the other 30 annotations. However, this was not the only driver for seemingly open cotton and soybean pangenomes. The rate of pangenome expansion *within* the seven Chu *et al.* [34] and 23 Liu *et al.* [33] genomes was over 6.3× and 2.1× faster in the original annotations than the IGC re-annotations respectively (Fig. 3C). Importantly, a portion of the expansion disparity between the two consortia also appears to be driven by biology: in the IGC re-annotated genomes, each Liu *et al.* genome contributed 1357 new HOGs, over double that of Chu *et al.*’s 677. This increased expansion rate may be due to the inclusion of diverse germplasm in Liu *et al.* but not Chu *et al.*

### Relative contributions of PAV categories across annotation methods

The structure of a pangenome is also often summarized by the degree and frequency of PAV, where ‘core’ sequences, which are found in all genomes, are compared to those that are in ‘private’ (found in a single genome), ‘cloud’ (< 50% presence), ‘shell’ (50–90% presence) and ‘near-core’ (>90% presence, missing in at least one genome) PAV categories. Like above, we compared a baseline expectation for the amount of sequence in PAV categories using k-mer frequencies from the reference genome assemblies to PAV categories from raw annotations and re-annotated genomes with IGC.

As expected from pangenome relatedness measures (Fig. 2B), PAV calculated from integration of raw gene annotations showed high levels of PAV (Fig. 3D and E) including obvious ‘bumps’ when switching between annotation consortia (Supplementary Fig. S2). The contribution to private gene groupings was particularly conspicuous: the number of private soybean genes was nearly three times higher in the original than IGC annotations. Thus, it is not surprising that PAV (as a function of membership in HOGs) was much more biased towards low frequency orthogroups in the raw than IGC annotations (Fig. 3D and E). For example, it was 3.56× (Fisher’s exact *P* <.001) more likely to observe low-frequency PAV orthogroups (not core or near-core) in raw than IGC soybean annotations. Importantly, ‘core’ genes were ∼2× more common in both species’ IGC re-annotated genomes than the originals. Thus, connecting orthologs and exploring candidate genes will be much more straightforward among these consistent annotations than the originals.

PAV among the IGC re-annotated genomes also more readily recapitulates biological expectations. Given sampling of more diverse material, we expected that low frequency HOGs would be much more common in the Liu *et al.* [33] set than Chu *et al.* [34]; indeed, this pattern was clear in the IGC re-annotated genomes where 41.0% of the Liu *et al.* HOGs were at < 50%, 4.6× higher than the 8.9% observed among Chu *et al.* genomes. However, these values were much closer in the original Liu *et al.* and Chu *et al.* annotations where 55.6% and 33.2% of genes had >50% absences respectively (Fig. 3F). Combined, the relatively enriched amount of high-frequency sequences in IGC annotations meshes far more closely with evolutionary expectations and sequence-level differences, indicating that both the apparent openness of pangenomes and the extent of PAV can be substantially influenced by annotation methodology.

We also compared patterns of PAV of IGC and raw annotations with Helixer [9, 22], a popular machine learning *ab initio* (and evidence-free) annotation program. Helixer-based annotations consistently yielded PAV profiles more similar to IGC than to raw annotations, characterized by increased core and reduced private HOG percentages (Table 1). We were surprised to observe that, despite a larger number of total orthogroups, annotation completeness, and false-positive statistics from Helixer were nearly identical to those of IGC—both statistics were uniformly much higher than the raw annotations hosted on external repositories and similar to IGC annotation statistics. However, we observed a concerning trend that has also been reported on the Helixer repository (https://github.com/usadellab/Helixer/issues/138): many genes (including several BUSCO markers) exhibit noncannonical gene structures. For example, across the 37 soybean Helixer annotations, on average, 16.4% of genes had internal coding exons shorter than 10 bp and 7.6% of genes had noncanonical splice sites (Supplementary Tables S5 and S6, and Table 1). To ensure that this enrichment of unlikely gene models is not specific to these more complex genomes, we also re-annotated the TAIR10 *A. thaliana* [37] genome with both Helixer and IGC and compared gene structures to the Araport11 [38] gold-standard annotation (Table 1). Similar to observations in cotton and soybean, Helixer produced a significantly higher percentage (9.9%) of noncanonical gene models compared to either IGC or Araport11 annotations, which had a much lower percentage (<1%) of such gene structures. Combined, these results point to the value of Helixer and likely other *ab initio* deep learning methods to harmonize annotations, but also potential problems when using unimproved Helixer annotations on their own.

**Table 1.** Counts of PAV type by species and annotation method

	Cotton – all			Soybean – all			Soybean – only Liu *et al.* [33]			*A. thaliana* TAIR10		
	Raw	Helixer	IGC	Raw	Helixer	IGC	Raw	Helixer	IGC	Araport11	Helixer	IGC
% core HOGs	21.9	59.1	58.2	4.9	20.5	21.7	16.0	21.6	22.7	.	.	.
% near-core	-	-	-	11.2	18.2	26.6	13.9	16.4	22.4	.	.	.
% shell	28.0	14.2	14.3	15.3	7.8	8.8	14.7	10.5	13.8	.	.	.
% cloud	21.7	11.1	8.4	32.5	24.2	18.2	25.2	23.4	17.7	.	.	.
% private	28.4	15.6	19.1	36.1	29.3	24.6	30.3	28.2	23.4	.	.	.
BUSCO %	98.0	98.9	99.8	96.7**	97.6	97.5	96.0	96.9	96.4	99.9	99.0	99.7
PSAURON	94.6	96.2	95.9	93.4**	96.6	95.6	93.5	96.4	95.5	95.2	97.1	96.5
% NC genes*	1.1	20.8	1.2	0.8	20.8	1.2	0.5	21.9	1.3	0.7	9.9	0.8

Numbers reported here are the percentage of statistics by HOGs for the original (‘raw’), IGC, and Helixer annotations. HOGs are classified by presence across genomes: core (100%), near-core (≥90%), shell (≥50%), cloud (<50% but > 1 genome), and private (single genome only). Near-core is not applicable for Cotton (*n* = 7) as no integer count falls between 90% and 100%. *NC = Noncanonical gene models, which are defined here as genes with splice sites other than GT-AG, GC-AG, or with internal coding exons 9bp in length or shorter. The numbers are averaged across all genomes in the pangenome set. See Supplementary Tables S5 and S6 for the numbers per genome and more granular breakdown of noncanonical events. **Averages exclude Zh13 [32], which is an older annotation with conspicuously low BUSCO scores.

### Quantifying the consistency of annotations

Our analyses of phylogenetic trees and pangenome composition strongly suggest that existing annotations contain high levels of artifactual variation. In many cases, the cause is clear. For example, sequence variation can lead to minor differences in annotation heuristics support scores causing stochasticity in borderline-quality genes presence–absence [39, 40]. However, in genomes like cotton with very little sequence variation, this artifact alone cannot explain the 6000+ private genes observed in many genomes.

**Figure 4.**
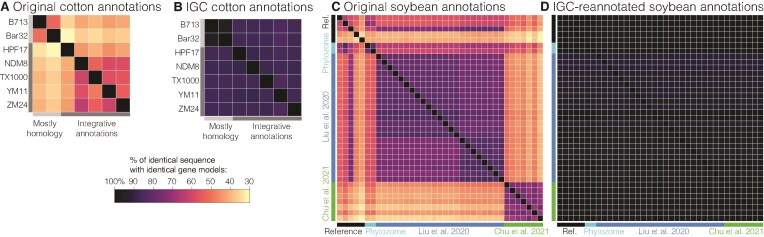
Consistency of annotations. (**A**) Gene models from each of the seven non-IGC cotton annotations (query, rows) were aligned to each other (target, columns) and the percent of the genes with exactly identical sequence that produced identical gene structures are reported. Row labels indicate variety names, while column labels indicate the type of annotation method used. (**B**) Shows the same statistic but for the external genomes re-annotated with the IGC pipeline. (**C**, **D**) Follow panels (A) and (B), respectively, except for all 37 of the soybean annotations. Row and column labels indicate the consortium that produced the original annotations: colored as in Fig. 2.

To better understand the proximate causes of gene annotation inconsistency, we examined loci with identical CDS between pairs of genomes and measured their predicted coordinate consistency and overlap (see the ‘Materials and methods’ section). We expected nearly all genomes and gene models to be consistent, given that these genes represent evolutionarily conserved, identical sequences. However, this was clearly not the case. In all comparisons, at least 13k genes mapped perfectly (with 31k on average), ensuring that our consistency analyses were based on a large subset of the protein-coding gene space; however, on average across all cotton genome pairs, only 44.5% of genes with identical DNA sequence had identical structure to the original gene predictions; HPF17-Bar32 was the extreme case where just 30.8% of identical sequences had identical gene model structure (Fig. 4A). Much of this inconsistency was attributable to differences in gene annotation methods, and consistency improved markedly with our IGC cotton annotations (from 44.5 to 87.0% agreement on average, Fig. 4A and B), although some discrepancies persisted. We observed similar patterns in soybean genomes (Fig. 4C and D) where the percentage of perfectly mapped genes with identical prediction in the target genome was 61.5% for original annotations versus 96.5% in our re-annotated set. While such structural differences may not affect PAV estimates, even small differences in predicted exon-intron structure can significantly impact downstream analyses of gene function, expression, and evolution.

It is notable that this trend persisted even when using the more relaxed overlap metric. Instead of requiring identical gene structures, we checked whether an identical coding DNA sequence produced any overlapping coordinates with a predicted gene in the target genome on the same strand. As expected under this relaxed criterion, agreement of raw annotations increased significantly in both cotton (44.5 → 80.9%) and soybean (61.5 → 89.3%). However, the IGC-reannotated consistency of cotton (87.0 → 97.7%) and soybean (96.5 → 99.1%) increased at a corresponding level (Supplementary Fig. S3).

While consistent annotation methodology dramatically reduced artifactual PAV, we also investigated whether using native RNA-seq (RNA extracted from the same genotype being annotated) affects annotation consistency. In controlled experiments with soybean YuDouNo_22, the annotation based on native RNA-seq, as opposed to RNA-seq from the reference cultivar Wm82, showed improved BUSCO completeness and PSAURON scores (Supplementary Table S7) but did not significantly affect the estimated PAV (Supplementary Fig. S4). This indicates that the source of RNA-seq data is not the major driver of the reduction in PAV we observed with consistent re-annotation. Supporting this conclusion, our cotton re-annotations, which all used native RNA-seq, showed similar magnitudes of PAV reduction compared to soybean, where native RNA-seq was not always available. These findings further emphasize that methodological consistency, rather than input data sources alone, is the primary factor in reducing artifactual PAV.

### Conclusions

The analyses presented here clearly demonstrate that annotation method matters when considering gene PAV. Beyond comparisons among qualitatively different approaches, which clearly swamp any biological signal of gene PAV, even consistent annotation methods can produce highly inflated estimates of pangenome ‘openness’. Such artifacts must be considered in all pangenome analysis. These results demonstrate that all analyses of pan-gene set frequencies should conduct explicit *post hoc* tests of whether a gene is truly present or absent. Furthermore, it is clear that, while tempting, applying a single *ab initio* approach does not resolve the issue of artifactual pangenome expansion. For example, when compared to evidence-based approaches, Helixer introduces elevated levels of gene PAV and annotates over 20-fold more genes with putatively noncanonical and biologically unrealistic gene models. These findings have profound implications for the interpretation of comparative genomics datasets: many studies draw evolutionary conclusions from gene content comparisons across genomes annotated by different groups, potentially leading to flawed interpretations about gene family evolution, adaptation, and species relationships. Our results highlight the critical importance of standardized annotation approaches when conducting multigenome comparisons and challenge the reliability of conclusions drawn from heterogeneously annotated genome collections.

## Supplementary Material

lqag011_Supplemental_Files

## Data Availability

All analysis scripts are available at https://github.com/tomasbruna/pangene-pav-integration, which retrieves all required data from Zenodo (https://zenodo.org/records/17902589).
