# Supplementary material for: Evolutionary and methodological considerations when interpreting gene presence–absence variation in pangenomes
Source: NAR Genom Bioinform. 2026 Jan 30;8(1):lqag011. doi: 10.1093/nargab/lqag011 (PMC12856356; doi:10.1093/nargab/lqag011)
Supplement: lqag011_Supplemental_Files [file lqag011_supplemental_files.zip › Bruna_Supplement.pdf]

Supplementary Material for: Evolutionary and methodological considerations when interpreting gene presence-absence variation in pangenomes

Tomáš Brůna[1*], Avinash Sreedasyam[1,2], Avril M. Harder[2], & John T. Lovell[1,2*]

[1]U.S. Department of Energy Joint Genome Institute,
Lawrence Berkeley National Laboratory, Berkeley, CA 94720, USA
[2]Genome Sequencing Center, HudsonAlpha Institute for Biotechnology, Huntsville, AL 35806, USA
*Corresponding authors: tbruna@lbl.gov, jlovell@hudsonalpha.org

Table of Contents

Supplementary Figures **2**
Supplementary Figure 1 2
Supplementary Figure 2 3
Supplementary Figure 3 4
Supplementary Figure 4 4
Supplementary Tables **5**
Supplementary Table 1 5
Supplementary Table 2 5
Supplementary Table 3 7
Supplementary Table 4 8
Supplementary Table 5 9
Supplementary Table 6 10
Supplementary Table 7 10
Supplementary References **11**

Supplementary Figures

Supplementary Figure 1

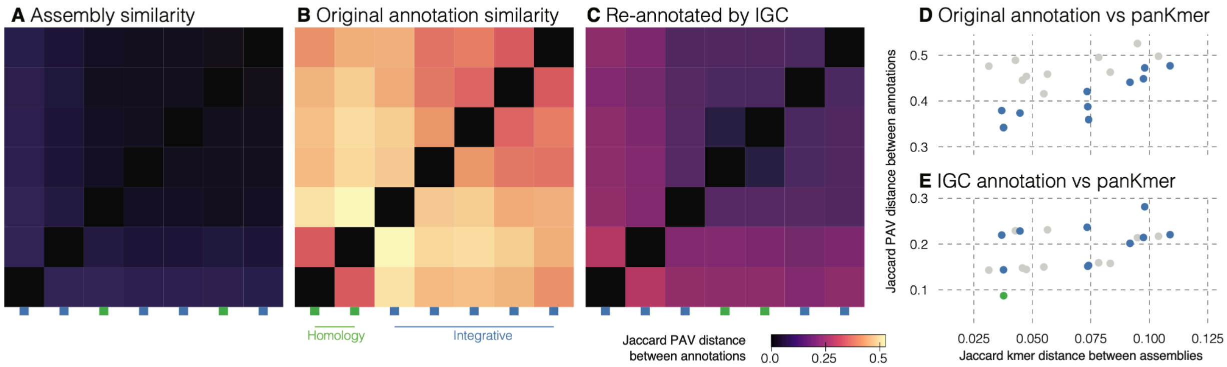

Supplementary Figure 1 | Cotton clustering by annotation and assembly similarity reveals that original gene PAV is driven almost exclusively by annotation method. All plots follow that of Figure 2, except with cotton data and no replication of genotypes. Distance matrices between cotton varieties were calculated from panKmer (A) and OrthoFinder gene presence-absence variation of the originally published (B) and IGC (C) protein-coding gene annotations. In each panel, distances are hierarchically clustered to group the most similar genomes together. The original annotation method type is flagged below each column in the matrices using colored bars: homology-based (green) and integrative (blue). The uniformly dark appearance of the panKmer matrix (A) reflects the high sequence similarity across all cotton genomes, in contrast to the structured patterns in (B). Correlations between the panKmer Jaccard distance and the original raw (D) and single-method IGC (E) PAV distances are presented where points are colored following panel B for comparisons within the same annotation method type, with grey indicating comparisons between different methods.

Supplementary Figure 2

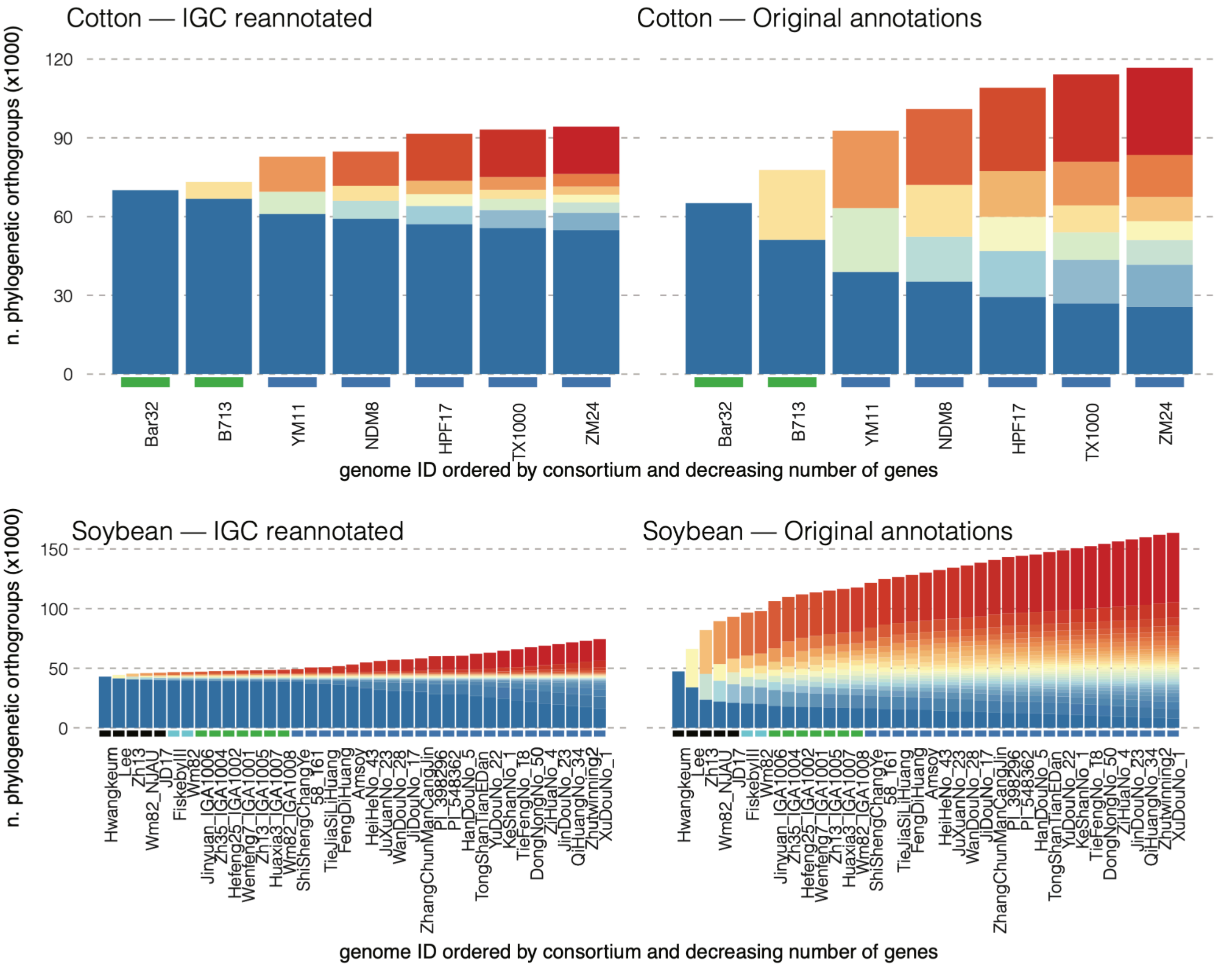

Supplementary Figure 2 | Pangenome expansion curves grouped by method type or consortium. This plot follows Fig. 3A-B, but with the annotations grouped by the original annotation method type (cotton) or consortium (soybean), then by decreasing numbers of annotated genes within each group. The annotation method type (cotton) or consortium (soybean) is labeled above the genome IDs using colored bars as in Supplementary Figure 1 and Figure 2, respectively. Colors indicate pangenome frequency categories, following the gradient in Figure 3 from blue (core, found in all genomes) to red (private, found in a single genome).

Supplementary Figure 3

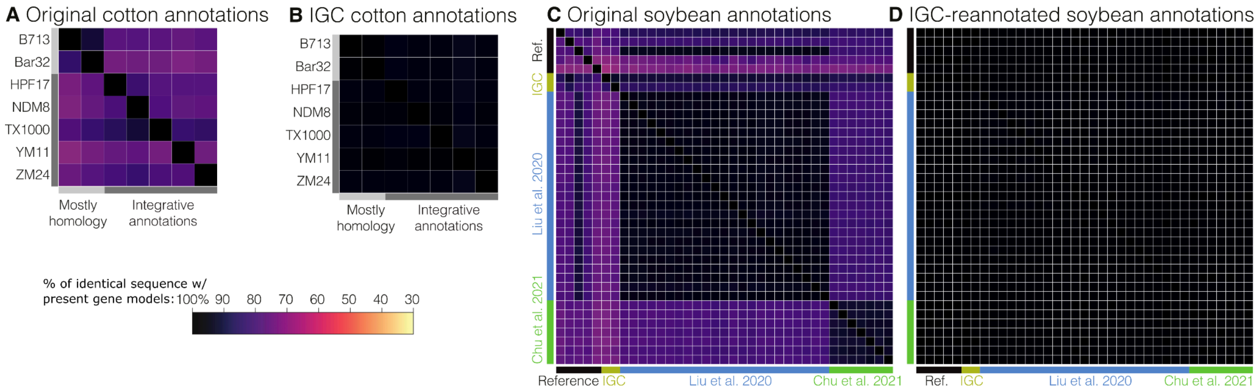

Supplementary Figure 3 | Consistency of annotations using the relaxed overlap metric. This figure is the counterpart to Figure 4 in the main text, but uses the relaxed "overlap" measure instead of exact match (see Methods for definition).

Supplementary Figure 4

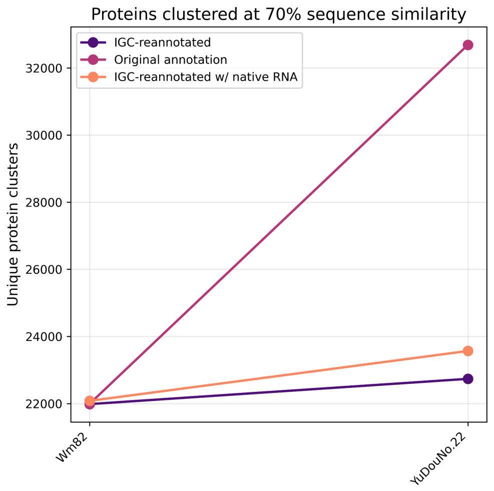

Supplementary Figure 4 | Effect of transcriptome choice in IGC annotation on proteins newly added to the pangenome set. Number of newly added proteins (at <70% sequence similarity to existing proteins) to the Wm82 reference set after adding proteins predicted in YuDouNo_22 using: (a) Existing Liu et al. (2020) annotation (*Original annotation*), (b) IGC annotations guided by the transcriptome used to generate the existing annotation from Liu et al. (2020) (*IGC-reannotated w/ native RNA*), and (c) IGC annotations guided by the Wm82 transcriptome (*IGC-reannotated*). Lines are ordered top to bottom as listed. Proteins were clustered using DIAMOND DeepClust (1).

Supplementary Tables

Supplementary Table 1

Supplementary Table 1 | Metadata for animal genomic comparisons in Figure 1.

Species	Common Name	NCBI URL
Chrysemys picta bellii	Western painted turtle	https://www.ncbi.nlm.nih.gov/datasets/genome/GCF_011386835.1/
Chelonia mydas	Green sea turtle	https://www.ncbi.nlm.nih.gov/datasets/genome/GCF_015237465.2/
Varanus komodoensis	Komodo dragon	https://www.ncbi.nlm.nih.gov/datasets/genome/GCF_004798865.1/
Alligator mississippiensis	American alligator	https://www.ncbi.nlm.nih.gov/datasets/genome/GCF_030867095.1/
Crocodylus porosus	Saltwater crocodile	https://www.ncbi.nlm.nih.gov/datasets/genome/GCF_001723895.1
Dromaius novaehollandiae	Emu	https://www.ncbi.nlm.nih.gov/datasets/genome/GCF_036370855.1
Struthio camelus	Ostrich	https://www.ncbi.nlm.nih.gov/datasets/genome/GCF_040807025.1/
Taeniopygia guttata	Zebra finch	https://www.ncbi.nlm.nih.gov/datasets/genome/GCF_048771995.1
Gallus gallus	Chicken	https://www.ncbi.nlm.nih.gov/datasets/genome/GCF_016699485.2/
Anolis carolinensis	Green anole lizard	https://www.ncbi.nlm.nih.gov/datasets/genome/GCF_035594765.1/
Pantherophis guttatus	Corn snake	https://www.ncbi.nlm.nih.gov/datasets/genome/GCF_029531705.1/
Ornithorhynchus anatinus	Platypus	https://www.ncbi.nlm.nih.gov/datasets/genome/GCF_004115215.2/
Bos taurus	Cattle	https://www.ncbi.nlm.nih.gov/datasets/genome/GCF_002263795.3/
Sus scrofa	Pig	https://www.ncbi.nlm.nih.gov/datasets/genome/GCF_000003025.6/
Mus musculus	House mouse	https://www.ncbi.nlm.nih.gov/datasets/genome/GCF_000001635.27/
Rattus norvegicus	Norway rat	https://www.ncbi.nlm.nih.gov/datasets/genome/GCF_036323735.1/
Gorilla gorilla	Western lowland gorilla	https://www.ncbi.nlm.nih.gov/datasets/genome/GCF_029281585.2/
Homo sapiens	Human	https://www.ncbi.nlm.nih.gov/datasets/genome/GCF_000001405.40/
Chrysemys picta bellii	Western painted turtle	https://www.ncbi.nlm.nih.gov/datasets/genome/GCF_011386835.1/
Chelonia mydas	Green sea turtle	https://www.ncbi.nlm.nih.gov/datasets/genome/GCF_015237465.2/

Supplementary Table 2

Supplementary Table 2 | Metadata for plant genomic comparisons in Figure 1.

Species	Common Name	NCBI URL
Cryptomeria japonica	Japanese Cedar	https://www.ncbi.nlm.nih.gov/datasets/genome/GCF_030272615.1/
Ananas comosus	Pineapple	https://www.ncbi.nlm.nih.gov/datasets/genome/GCF_001540865.1/
Brachypodium distachyon	Brachypodium	https://www.ncbi.nlm.nih.gov/datasets/genome/GCF_000005505.3/
Musa acuminata	Banana	https://www.ncbi.nlm.nih.gov/datasets/genome/GCF_036884655.1/
Oryza sativa	Rice	https://www.ncbi.nlm.nih.gov/datasets/genome/GCF_034140825.1/
Sorghum bicolor	Sorghum	https://www.ncbi.nlm.nih.gov/datasets/genome/GCF_000003195.3/
Setaria viridis	Green foxtail millet	https://www.ncbi.nlm.nih.gov/datasets/genome/GCF_005286985.2/
Zea mays	Maize	https://www.ncbi.nlm.nih.gov/datasets/genome/GCF_902167145.1/
Telopea speciosissima	Telopea speciosissima	https://www.ncbi.nlm.nih.gov/datasets/genome/GCF_018873765.1/
Solanum lycopersicum	Tomato	https://www.ncbi.nlm.nih.gov/datasets/genome/GCF_036512215.1/
Coffea arabica	Arabica coffee	https://www.ncbi.nlm.nih.gov/datasets/genome/GCF_036785885.1/
Vitis vinifera	Grapevine	https://www.ncbi.nlm.nih.gov/datasets/genome/GCF_030704535.1/
Malus domestica	Apple	https://www.ncbi.nlm.nih.gov/datasets/genome/GCF_042453785.1/
Manihot esculenta	Cassava	https://www.ncbi.nlm.nih.gov/datasets/genome/GCF_001659605.2/
Phaseolus vulgaris	Common bean	https://www.ncbi.nlm.nih.gov/datasets/genome/GCF_000499845.2/
Populus trichocarpa	Black cottonwood	https://www.ncbi.nlm.nih.gov/datasets/genome/GCF_000002775.5/
Prunus persica	Peach	https://www.ncbi.nlm.nih.gov/datasets/genome/GCF_000346465.2/
Rosa chinensis	Chinese rose	https://www.ncbi.nlm.nih.gov/datasets/genome/GCF_002994745.2/
Glycine max	Soybean	https://www.ncbi.nlm.nih.gov/datasets/genome/GCF_000004515.6/
Carya illinoinensis Pawnee	Pecan	https://www.ncbi.nlm.nih.gov/datasets/genome/GCF_018687715.1/
Citrus sinensis	Sweet orange	https://www.ncbi.nlm.nih.gov/datasets/genome/GCF_022201045.2/
Brassica rapa	Brapa	https://www.ncbi.nlm.nih.gov/datasets/genome/GCF_000309985.2/
Arabidopsis thaliana	Thale cress	https://www.ncbi.nlm.nih.gov/datasets/genome/GCF_000001735.4/

Supplementary Table 3

Supplementary Table 3 | Metadata for 37 soybean genomes. Source information from Soybase and assembly/annotation statistics calculated from the raw genomes are presented.

Genotype ID	Consortium	Source	Assembly Size (Mb)	Assembly contig N50 (Mb)	Origi. n. genes	Orig. BUSCO	Orig PSAURON	IGC n. genes	IGC BUSCO	IGC PSAURON	Helixer n. genes	Helixer BUSCO	Helixer PSAURON
JD17	ref	Jidou 17 is an Chinese elite cultivar, widely grown in central China (in maturity group III). This variety is recognized for high yields and tolerance for high planting densities.	995.3	18	52,840	98.5	94.9	46,101	99.6	95.9	49,370	98.8	97.0
Wm82_NJAU	ref	Williams 82 sequenced for T2T assembly of Wang, Zhang et al. 2023	1,011.8	51.2	55,497	99.4	90.5	45,966	99.5	95.9	49,112	98.9	97.0
Zh13	ref	Zhonghuang 13 is a Chinese cultivar derived from accessions Yudou 18 and Zhongzuo 90052-76 by pedigree selection for high yield and stress tolerance (Shen et al., 2018; https://doi.org/10.1007/s11427-018-9360-0).	1,011.8	18	55,573	81.5	76.8	46,292	99.4	95.7	49,800	98.7	96.8
Hwangkeum	ref	Hwangkeum is an important South Korean cultivar, released in 1979 and widely used since then as a breeding parent in Korea. Hwangkeum has a determinate growth habit and nonshattering pods and is adapted to the middle Korean peninsula (Maturity Group V).	933.1	7.8	58,570	96.8	94.7	45,766	99.3	95.9	48,978	98.8	96.9
Lee	ref	Cultivar Lee, which derives from a cross of Chinese lines CNS and S-100, has been widely used as a parent in many breeding projects in the Southern U.S. and in Brazil (Wysmierski and Vello, 2013; http://doi.org/10.1590/S1415-47572013005000041). The variety is notable for resistance to Phytophthora rot, Peanut Mottle Virus, and bacterial pustule (Wysmierski and Vello, 2013).	1,016.4	32.2	56,725	96.6	93.4	45,864	99.4	95.9	49,178	98.8	97.0
Wm82	Phytozome	Williams 82 was derived from backcrossing a phytophthora root rot resistance locus from the donor parent Kingwa into the recurrent parent Williams. A sub-line, Wm82-ISU-01, was derived by Robert Stupar (University of Minnesota) by inbreeding original W82 seed. That sub-line, registered as PI 704477, was used for assembly Wm82.gnm6 / Wm82.a6.	1,011.1	44.4	48,387	99.5	94.6	45,952	99.5	95.9	49,058	98.9	97.0
FiskebyIII	Phytozome	Bred by the late Dr. Sven Holmberg in Fiskeby, Sweden. Highly nutritious ,Äî up to 40% protein, high in calcium, iron, and vitamins (particularly A, B1, B12, and C). Thrives in northern climates. 75-80 days.	992.2	15.7	52,783	99.7	93.3	46,024	99.5	95.9	49,451	98.9	96.9
XuDouNo_1	Liu2020	Cultivar Xu Dou No.1 (SoyC01 from China, Jiang Su, from Liu et al. 2020	1,003.8	23.5	54,405	92.4	93.4	44,445	91.1	95.0	53,297	93.6	95.7
Zhutwinning2	Liu2020	Landrace Zhutwinning2 (SoyL01 from China, from Liu et al. 2020	999.2	23.3	54,502	93.0	93.4	44,549	91.3	95.0	53,323	93.4	95.8
QiHuangNo_34	Liu2020	Cultivar Qi Huang No.34 (SoyC08 from China, Shan Dong, from Liu et al. 2020	1,002.3	22.4	54,747	93.8	93.4	44,932	92.9	95.3	52,437	94.9	95.9
JinDouNo_23	Liu2020	Cultivar Jin Dou No.23 (SoyC07 from China, Shan Xi, from Liu et al. 2020	1,008.8	21.8	54,792	94.2	93.3	44,937	93.7	95.2	52,251	95.4	95.9
ZiHuaNo_4	Liu2020	Landrace Zi Hua No.4 (SoyL02 from China, Hei Long Jiang, from Liu et al. 2020	1,011.7	23.1	54,803	94.2	93.4	45,060	93.9	95.2	51,865	95.4	96.1
DongNongNo_50	Liu2020	Cultivar Dong Nong No.50 (SoyC12 from China, Hei Long Jiang, from Liu et al. 2020	1,025.1	20	55,000	94.7	93.5	45,136	94.4	95.2	53,009	95.8	96.0
TieFengNo_18	Liu2020	Cultivar Tie Feng No.18 (SoyC02 from China, Hei Long Jiang, from Liu et al. 2020	1,011.1	23.8	55,191	95.6	93.5	45,513	96.1	95.3	51,470	96.6	96.3
KeShanNo_1	Liu2020	Cultivar Ke Shan No.1 (SoyC14 from China, Hei Long Jiang, from Liu et al. 2020	1,007.4	23	55,267	94.9	93.4	45,613	94.5	95.2	52,058	95.6	96.1
YuDouNo_22	Liu2020	Cultivar Yu Dou No.22 (SoyC06 from China, He Nan, from Liu et al. 2020	1,007.9	23	55,619	96.3	93.6	45,712	96.6	95.5	50,893	97.1	96.5
TongShanTianEDan	Liu2020	Landrace Tong Shan Tian E Dan (SoyL03 from China, Jiang Su, from Liu et al. 2020	1,039.5	21.3	55,769	95.5	93.5	45,878	95.2	95.4	52,925	95.9	96.2
HanDouNo_5	Liu2020	Cultivar Han Dou No.5 (SoyC09 from China, He Bei, from Liu et al. 2020	1,004.1	22.6	55,926	98.3	93.7	46,043	99.5	95.8	49,499	98.9	96.9
PI_548362	Liu2020	Cultivar PI 548362 (SoyC10 from United States, Illinois, from Liu et al. 2020	1,004.9	19.8	56,011	98.0	93.4	46,123	99.5	95.7	49,424	98.7	96.8
PI_398296	Liu2020	Landrace PI 398296 (SoyL05 from South Korea, Kyonggi, from Liu et al. 2020	1,059.8	22.1	56,086	95.6	93.4	46,038	95.4	95.4	53,066	96.4	96.1
ZhangChunManCang Jin	Liu2020	Landrace Chang Chun Man Cang Jin (SoyL06 from China, Ji Lin, from Liu et al. 2020	997.8	21.5	56,573	97.4	93.1	45,766	98.0	95.7	50,345	97.8	96.7
JiDouNo_17	Liu2020	Cultivar Ji Dou No.17 (SoyC11 from China, He Bei, from Liu et al. 2020	1,024.5	18.8	56,750	97.8	93.7	46,612	99.2	95.7	50,803	98.7	96.7
WanDouNo_28	Liu2020	Cultivar Wan Dou No.28 (SoyC04 from China, An Hui, from Liu et al. 2020	1,001.5	22.7	57,777	96.8	93.8	45,692	98.0	95.7	50,232	97.9	96.6
JuXuanNo_23	Liu2020	Cultivar Ju Xuan No.23 (SoyC03 from China, Shan Dong, from Liu et al. 2020	1,007.8	23.9	57,792	96.2	93.7	45,562	97.6	95.7	50,588	97.3	96.5
HeiHeNo_43	Liu2020	Cultivar Hei He No.43 (SoyC13 from China, Hei Long Jiang, from Liu et al. 2020	1,010.1	23.8	57,976	95.6	93.5	45,820	96.6	95.4	51,445	97.2	96.2
Amsoy	Liu2020	Cultivar Amsoy (SoyC05 from United States, Iowa, from Liu et al. 2020	992.3	22.7	58,040	96.8	93.7	45,849	97.6	95.7	50,399	97.8	96.7

FengDiHuang	Liu2020	Landrace Feng Di Huang (SoyL07 from China, Ji Lin, from Liu et al. 2020	1,004.7	22.9	58,259	97.1	93.7	45,858	98.4	95.7	50,036	98.1	96.7
TieJiaSiLiHuang	Liu2020	Landrace Tie Jia Si Li Huang (SoyL08 from China, Ji Lin, from Liu et al. 2020	999.7	22.6	58,496	97.9	93.6	46,137	99.4	95.7	49,263	98.8	96.8
58_161	Liu2020	Landrace 58-161 (SoyL04 from China, Jiang Su, from Liu et al. 2020	1,005.2	21.8	58,881	96.7	93.7	45,776	97.8	95.6	50,730	97.9	96.4
ShiShengChangYe	Liu2020	Landrace Shi Sheng Chang Ye (SoyL09 from Japan, Hokkaido, from Liu et al. 2020	1,028.2	23.1	59,588	98.3	93.7	47,290	99.5	95.7	51,482	98.9	96.6
Wm82_IGA1008	Chu2021	Williams 82, the soybean cultivar used to produce the reference genome sequence, was derived from backcrossing a phytophthora root rot resistance locus from the donor parent Kingwa into the recurrent parent Williams. This line, IGA1008, was sequenced and annotated by Chu, et al. (2021).	993.0	1.9	57,286	98.1	92.5	46,221	99.5	95.8	49,476	98.8	96.9
Huaxia3_IGA1007	Chu2021	Glycine max Huaxia 3 (IGA1007) was sequenced and annotated by Chu, et al. (2021).	986.0	6.2	57,393	97.5	92.5	46,094	99.5	95.7	49,415	98.9	96.9
Zh13_IGA1005	Chu2021	Zhonghuang 13 is a Chinese cultivar derived from accessions Yudou 18 and Zhongzuo 90052-76 by pedigree selection for high yield and stress tolerance (Shen et al., 2018; https://doi.org/10.1007/s11427-018-9360-0). This line, IGA1005, was sequenced and annotated by Chu, et al. (2021).	988.8	4.7	57,474	97.5	92.6	46,085	99.4	95.8	49,329	98.8	96.8
Wenfeng7_IGA1001	Chu2021	Glycine max Wenfeng 7 (IGA1001) was sequenced and annotated by Chu, et al. (2021).	996.7	1.7	57,505	97.4	92.8	46,205	99.4	95.8	49,739	98.8	96.9
Hefeng25_IGA1002	Chu2021	Glycine max Hefeng 25 (IGA1002) was sequenced and annotated by Chu, et al. (2021).	987.3	2.9	58,102	97.3	92.6	46,205	99.5	95.8	49,699	98.9	96.8
Zh35_IGA1004	Chu2021	Glycine max Zhonghuang 35 (IGA1004) was sequenced and annotated by Chu, et al. (2021).	1,001.3	1.4	58,150	97.7	92.8	46,613	99.3	95.8	50,711	98.8	96.8
Jinyuan_IGA1006	Chu2021	Glycine max Jinyuan (IGA1006) was sequenced and annotated by Chu, et al. (2021).	995.7	4.3	58,392	96.9	92.4	46,131	99.5	95.8	49,428	98.9	96.9

Supplementary Table 4

Supplementary Table 4 | Metadata for 7 cotton genomes. Source information from Cottongen and assembly/annotation statistics calculated from the raw genomes are presented.

Genotype ID	Consortium	Source	Assembly Size (Mb)	Assembly contig N50 (Mb)	Orig. n. genes	Orig. BUSCO	Orig PSAURON	IGC n. genes	IGC BUSCO	IGC PSAURON	Helixer n. genes	Helixer BUSCO	Helixer PSAURON
ZM24	n/a	G. hirsutum acc. ZM24 was sequenced and annotated by Yang et al., 2019.	2,308.2	1.9	73,707	99.5	95.2	70,882	99.8	96.1	77,660	98.9	96.2
YM11	n/a	G. hirsutum YM11, an accession with elite cold tolerance was sequenced and annotated by Wang et al., 2024.	2,343.1	88.9	84,720	98.5	91.0	76,335	99.7	95.6	84,916	99.0	95.8
TX1000	n/a	G. hirsutum race punctatum accession no. Punctatum 25 (TX-1000) was sequenced and annotated by Peng et al., 2022.	2,292.5	11.4	74,520	97.7	95.4	70,997	99.8	96.0	78,061	99.0	96.3
NDM8	n/a	G. hirsutum NDM8 was sequenced and annotated by Ma et al., 2021.	2,291.8	13.1	79,729	99.0	93.5	70,248	99.8	96.2	77,211	99.1	96.3
HPF17	n/a	G. purpurascens HPF17 is a primitive race of G. hirsutum was sequenced and annotated by Cheng et al., 2024	2,558.7	10.1	79,146	97.5	95.1	79,426	99.8	95.4	87,197	98.9	95.9
Bar32	USDA	G. hirsutum Bar32 is a nematode-resistant line sequenced and annotated by Perkin et al., 2021.	2454.3	75.3	75,988	97.8	95.4	70,881	99.8	96.0	77,316	98.8	96.4
B713	USDA	G. hirsutum B713 is a nematode-resistant line sequenced and annotated by Perkin et al., 2021.	2,296.1	68.8	71,531	96.0	96.3	70,816	99.8	96.1	77,427	98.9	96.4

Supplementary Table 5

Supplementary Table 5 | Detailed statistics of non-canonical events in gene models across the soybean genomes. Each statistic shows the absolute count of occurrences, and the percentage indicates the proportion of unique genes affected.

Genotype ID	Original annotation				IGC annotation				Helixer annotation			
	Short internal coding exons (<10 bp)	Ultra-short internal coding exons (<3 bp)	Non-canonical introns	Genes with any Issues	Short internal coding exons (<10 bp)	Ultra-short internal coding exons (<3 bp)	Non-canonical introns	Genes with any Issues	Short internal coding exons (<10 bp)	Ultra-short internal coding exons (<3 bp)	Non-canonical introns	Genes with any Issues
JD17	157 (0.3%)	0 (0.0%)	121 (0.2%)	272 (0.5%)	134 (0.3%)	10 (0.0%)	304 (0.7%)	430 (0.9%)	10,215 (15.2%)	7,790 (12.3%)	3,518 (6.5%)	9,333 (18.9%)
Wm82_NJAU	128 (0.2%)	6 (0.0%)	343 (0.6%)	503 (0.9%)	128 (0.3%)	10 (0.0%)	272 (0.6%)	392 (0.8%)	10,185 (15.1%)	7,852 (12.4%)	3,403 (6.3%)	9,188 (18.7%)
Zh13	150 (0.3%)	8 (0.0%)	147 (0.2%)	273 (0.5%)	139 (0.3%)	13 (0.0%)	363 (0.8%)	491 (1.1%)	10,360 (15.2%)	7,927 (12.4%)	3,540 (6.5%)	9,426 (18.9%)
Hwangkeum	193 (0.3%)	0 (0.0%)	182 (0.3%)	366 (0.6%)	130 (0.3%)	12 (0.0%)	355 (0.8%)	472 (1.0%)	10,089 (15.1%)	7,727 (12.3%)	3,461 (6.5%)	9,218 (18.8%)
Lee	676 (1.1%)	1 (0.0%)	0 (0.0%)	651 (1.1%)	130 (0.3%)	10 (0.0%)	315 (0.7%)	437 (0.9%)	10,053 (15.0%)	7,735 (12.2%)	3,398 (6.3%)	9,119 (18.5%)
Wm82	115 (0.2%)	4 (0.0%)	322 (0.6%)	423 (0.9%)	128 (0.3%)	10 (0.0%)	268 (0.6%)	387 (0.8%)	10,200 (15.2%)	7,807 (12.5%)	3,417 (6.3%)	9,256 (18.9%)
FiskebyIII	148 (0.3%)	9 (0.0%)	502 (0.8%)	584 (1.1%)	131 (0.3%)	11 (0.0%)	352 (0.8%)	473 (1.0%)	10,232 (15.1%)	7,907 (12.5%)	3,508 (6.5%)	9,299 (18.8%)
XuDouNo_1	241 (0.4%)	11 (0.0%)	46 (0.1%)	281 (0.5%)	182 (0.4%)	31 (0.1%)	607 (1.3%)	768 (1.7%)	14,991 (20.1%)	11,929 (17.0%)	6,553 (10.7%)	13,811 (25.9%)
Zhutwinning2	237 (0.4%)	17 (0.0%)	61 (0.1%)	288 (0.5%)	195 (0.4%)	32 (0.1%)	574 (1.3%)	748 (1.7%)	14,743 (19.8%)	11,803 (16.9%)	6,419 (10.5%)	13,581 (25.5%)
QiHuangNo_34	239 (0.4%)	15 (0.0%)	44 (0.1%)	279 (0.5%)	177 (0.4%)	26 (0.1%)	585 (1.3%)	744 (1.7%)	13,902 (19.1%)	11,092 (16.3%)	5,926 (10.0%)	12,941 (24.7%)
JinDouNo_23	238 (0.4%)	11 (0.0%)	49 (0.1%)	281 (0.5%)	174 (0.4%)	27 (0.1%)	554 (1.2%)	717 (1.6%)	13,670 (18.9%)	10,830 (15.9%)	5,864 (9.9%)	12,763 (24.4%)
ZiHuaNo_4	238 (0.4%)	12 (0.0%)	51 (0.1%)	277 (0.5%)	165 (0.3%)	17 (0.0%)	576 (1.3%)	723 (1.6%)	13,495 (18.6%)	10,574 (15.6%)	5,655 (9.6%)	12,458 (24.0%)
DongNongNo_50	248 (0.4%)	13 (0.0%)	46 (0.1%)	287 (0.5%)	186 (0.4%)	25 (0.1%)	558 (1.2%)	728 (1.6%)	13,992 (19.1%)	11,070 (16.0%)	5,989 (9.9%)	13,023 (24.6%)
TieFengNo_18	213 (0.4%)	11 (0.0%)	47 (0.1%)	256 (0.5%)	142 (0.3%)	15 (0.0%)	452 (1.0%)	586 (1.3%)	12,537 (17.7%)	9,841 (14.8%)	5,018 (8.8%)	11,663 (22.7%)
KeShanNo_1	229 (0.4%)	14 (0.0%)	54 (0.1%)	272 (0.5%)	158 (0.3%)	24 (0.1%)	529 (1.1%)	672 (1.5%)	12,930 (18.1%)	10,207 (15.2%)	5,457 (9.3%)	12,118 (23.3%)
YuDouNo_22	192 (0.3%)	10 (0.0%)	38 (0.1%)	229 (0.4%)	146 (0.3%)	13 (0.0%)	462 (1.0%)	592 (1.3%)	11,857 (16.8%)	9,237 (13.9%)	4,529 (8.0%)	10,860 (21.3%)
TongShanTianEDan	220 (0.4%)	9 (0.0%)	53 (0.1%)	266 (0.5%)	158 (0.3%)	23 (0.1%)	522 (1.1%)	666 (1.4%)	13,375 (18.1%)	10,543 (15.2%)	5,459 (9.2%)	12,292 (23.2%)
HanDouNo_5	162 (0.3%)	9 (0.0%)	39 (0.1%)	196 (0.3%)	137 (0.3%)	11 (0.0%)	344 (0.7%)	467 (1.0%)	10,378 (15.2%)	7,945 (12.4%)	3,550 (6.5%)	9,376 (18.9%)
PI_548362	179 (0.3%)	7 (0.0%)	44 (0.1%)	219 (0.4%)	134 (0.3%)	8 (0.0%)	316 (0.7%)	442 (1.0%)	10,352 (15.3%)	7,962 (12.5%)	3,455 (6.4%)	9,349 (18.9%)
PI_398296	230 (0.4%)	10 (0.0%)	41 (0.1%)	263 (0.5%)	175 (0.4%)	20 (0.0%)	527 (1.1%)	688 (1.5%)	13,299 (18.2%)	10,480 (15.2%)	5,502 (9.2%)	12,436 (23.4%)
ZhangChunManCangJin	190 (0.3%)	6 (0.0%)	41 (0.1%)	223 (0.4%)	140 (0.3%)	9 (0.0%)	393 (0.8%)	524 (1.1%)	11,225 (16.1%)	8,722 (13.4%)	4,246 (7.6%)	10,289 (20.4%)
JiDouNo_17	204 (0.3%)	9 (0.0%)	42 (0.1%)	240 (0.4%)	150 (0.3%)	16 (0.0%)	339 (0.7%)	481 (1.0%)	10,969 (15.7%)	8,431 (12.8%)	3,782 (6.8%)	9,917 (19.5%)
WanDouNo_28	216 (0.4%)	13 (0.0%)	52 (0.1%)	263 (0.5%)	164 (0.3%)	17 (0.0%)	409 (0.9%)	552 (1.2%)	11,207 (16.3%)	8,767 (13.6%)	4,118 (7.4%)	10,299 (20.5%)
JuXuanNo_23	210 (0.4%)	17 (0.0%)	32 (0.1%)	237 (0.4%)	145 (0.3%)	19 (0.0%)	469 (1.0%)	597 (1.3%)	11,502 (16.5%)	8,898 (13.6%)	4,455 (7.9%)	10,597 (20.9%)
HeiHeNo_43	223 (0.4%)	12 (0.0%)	47 (0.1%)	264 (0.5%)	173 (0.4%)	24 (0.1%)	485 (1.0%)	641 (1.4%)	12,309 (17.3%)	9,646 (14.4%)	4,878 (8.4%)	11,345 (22.1%)
Amsoy	215 (0.4%)	16 (0.0%)	33 (0.1%)	243 (0.4%)	143 (0.3%)	13 (0.0%)	431 (0.9%)	562 (1.2%)	11,193 (16.2%)	8,668 (13.4%)	4,286 (7.7%)	10,451 (20.7%)
FengDiHuang	199 (0.3%)	16 (0.0%)	53 (0.1%)	246 (0.4%)	141 (0.3%)	13 (0.0%)	405 (0.9%)	532 (1.2%)	10,959 (15.9%)	8,421 (13.1%)	4,025 (7.3%)	10,093 (20.2%)
TieJiaSiLiHuang	197 (0.3%)	12 (0.0%)	49 (0.1%)	242 (0.4%)	144 (0.3%)	15 (0.0%)	345 (0.7%)	478 (1.0%)	10,282 (15.2%)	7,923 (12.4%)	3,464 (6.4%)	9,299 (18.9%)
58_161	219 (0.4%)	8 (0.0%)	50 (0.1%)	264 (0.5%)	145 (0.3%)	15 (0.0%)	436 (0.9%)	565 (1.2%)	11,549 (16.6%)	8,934 (13.6%)	4,333 (7.7%)	10,645 (21.0%)
ShiShengChangYe	186 (0.3%)	14 (0.0%)	35 (0.1%)	218 (0.4%)	148 (0.3%)	17 (0.0%)	368 (0.8%)	502 (1.1%)	11,042 (15.6%)	8,483 (12.7%)	3,849 (6.7%)	10,035 (19.5%)
Wm82_IGA1008	120 (0.2%)	5 (0.0%)	1,220 (1.8%)	1,183 (2.1%)	131 (0.3%)	10 (0.0%)	276 (0.6%)	398 (0.9%)	10,414 (15.3%)	7,977 (12.6%)	3,485 (6.4%)	9,401 (19.0%)
Huaxia3_IGA1007	151 (0.2%)	5 (0.0%)	933 (1.4%)	998 (1.7%)	136 (0.3%)	15 (0.0%)	341 (0.7%)	465 (1.0%)	10,281 (15.1%)	7,893 (12.3%)	3,430 (6.3%)	9,249 (18.7%)
Zh13_IGA1005	139 (0.2%)	5 (0.0%)	1,007 (1.5%)	1,051 (1.8%)	138 (0.3%)	14 (0.0%)	352 (0.8%)	476 (1.0%)	10,201 (15.0%)	7,793 (12.3%)	3,467 (6.4%)	9,274 (18.8%)
Wenfeng7_IGA1001	142 (0.2%)	7 (0.0%)	992 (1.5%)	1,037 (1.8%)	143 (0.3%)	13 (0.0%)	354 (0.8%)	486 (1.1%)	10,490 (15.3%)	7,979 (12.5%)	3,540 (6.5%)	9,465 (19.0%)
Hefeng25_IGA1002	135 (0.2%)	6 (0.0%)	1,045 (1.6%)	1,089 (1.9%)	148 (0.3%)	14 (0.0%)	351 (0.8%)	489 (1.1%)	10,350 (15.1%)	7,884 (12.3%)	3,453 (6.4%)	9,292 (18.7%)
Zh35_IGA1004	135 (0.2%)	8 (0.0%)	999 (1.5%)	1,044 (1.8%)	145 (0.3%)	21 (0.1%)	369 (0.8%)	499 (1.1%)	10,784 (15.4%)	8,289 (12.7%)	3,763 (6.7%)	9,783 (19.3%)
Jinyuan_IGA1006	143 (0.2%)	6 (0.0%)	1,074 (1.6%)	1,104 (1.9%)	137 (0.3%)	9 (0.0%)	335 (0.7%)	461 (1.0%)	10,156 (14.9%)	7,743 (12.2%)	3,414 (6.3%)	9,192 (18.6%)

Supplementary Table 6

Supplementary Table 6 | Detailed statistics of non-canonical events in gene models across the cotton genomes. Each statistic shows the absolute count of occurrences, and the percentage indicates the proportion of unique genes affected.

Genotype ID	Original annotation				IGC annotation				Helixer annotation			
	Short internal coding exons (<10 bp)	Ultra-short internal coding exons (<3 bp)	Non-canonical introns	Genes with any Issues	Short internal coding exons (<10 bp)	Ultra-short internal coding exons (<3 bp)	Non-canonical introns	Genes with any Issues	Short internal coding exons (<10 bp)	Ultra-short internal coding exons (<3 bp)	Non-canonical introns	Genes with any Issues
ZM24	141 (0.2%)	0 (0.0%)	177 (0.2%)	316 (0.4%)	200 (0.3%)	35 (0.1%)	769 (0.9%)	858 (1.2%)	16,897 (15.6%)	12,446 (12.3%)	7,222 (8.4%)	16,155 (20.8%)
YM11	382 (0.4%)	0 (0.0%)	90 (0.1%)	457 (0.5%)	184 (0.2%)	33 (0.0%)	821 (0.9%)	885 (1.2%)	17,909 (15.2%)	13,067 (12.0%)	8,170 (8.8%)	17,762 (20.9%)
TX1000	288 (0.4%)	14 (0.0%)	2,363 (2.4%)	2,042 (2.7%)	180 (0.2%)	27 (0.0%)	694 (0.8%)	771 (1.1%)	16,677 (15.4%)	12,242 (12.2%)	7,172 (8.3%)	16,053 (20.6%)
NDM8	91 (0.1%)	1 (0.0%)	177 (0.2%)	264 (0.3%)	181 (0.2%)	25 (0.0%)	754 (0.9%)	826 (1.2%)	16,884 (15.6%)	12,432 (12.4%)	7,066 (8.2%)	16,009 (20.7%)
HPF17	116 (0.1%)	0 (0.0%)	45 (0.1%)	159 (0.2%)	199 (0.2%)	31 (0.0%)	864 (0.9%)	934 (1.2%)	18,868 (15.4%)	13,724 (12.1%)	8,288 (8.6%)	18,200 (20.9%)
Bar32	721 (0.9%)	0 (0.0%)	3 (0.0%)	2,561 (3.4%)	191 (0.3%)	28 (0.0%)	807 (1.0%)	895 (1.3%)	16,791 (15.5%)	12,332 (12.3%)	7,155 (8.4%)	15,992 (20.7%)
B713	99 (0.1%)	0 (0.0%)	2 (0.0%)	94 (0.1%)	171 (0.2%)	26 (0.0%)	810 (1.0%)	882 (1.2%)	16,885 (15.6%)	12,458 (12.4%)	7,113 (8.3%)	16,089 (20.8%)

Supplementary Table 7

Supplementary Table 7 | Effect of transcriptome choice in IGC annotation on BUSCO completeness and PSAURON score. BUSCO completeness and PSAURON scores are shown for: (a) IGC annotations guided by the Wm82 transcriptome, (b) IGC annotations guided by the transcriptome used to generate the existing annotation from Liu et al. (2020), and (c) the existing Liu et al. (2020) annotation.

Genotype ID	Consortium	Origi. annotated genes (n)	Orig. BUSCO (%)	Orig pSAURON	IGC annotated genes (n)	IGC BUSCO (%)	IGC PSAURON	BUSCO Improvement	PSAURON improvement
YuDouNo_22: IGC w/ transcriptome from Wm82	Liu2020	55,619	96.3	93.6	45,712	96.6	95.5	0.3	1.9
YuDouNo_22: IGC w/ native transcriptome from Liu et al. (2020)					45,835	**97.8**	**96.4**	**1.5**	**2.8**

Supplementary References

1. Buchfink,B., Ashkenazy,H., Reuter,K., Kennedy,J.A. and Drost,H.-G. (2023) Sensitive clustering of protein sequences at tree-of-life scale using DIAMOND DeepClust. *bioRxiv*, 10.1101/2023.01.24.525373.
